# Intriguing electronic, optical and photocatalytic performance of BSe, M_2_CO_2_ monolayers and BSe–M_2_CO_2_ (M = Ti, Zr, Hf) van der Waals heterostructures

**DOI:** 10.1039/d1ra07569a

**Published:** 2021-12-21

**Authors:** M. Munawar, M. Idrees, Iftikhar Ahmad, H. U. Din, B. Amin

**Affiliations:** Department of Physics, Abbottabad University of Science & Technology Abbottabad 22010 Pakistan binukhn@gmail.com; Center for Computational Materials Science, University of Malakand Chakdara 18800 Pakistan; Department of Physics, Gomal University DI Khan Pakistan; Department of Physics, Bacha Khan University Charsadda Pakistan

## Abstract

Using density functional (DFT) theory calculations, we have investigated the electronic band structure, optical and photocatalytic response of BSe, M_2_CO_2_ (M = Ti, Zr, Hf) monolayers and their corresponding BSe–M_2_CO_2_ (M = Ti, Zr, Hf) van der Waals (vdW) heterostructures. Optimized lattice constant, bond length, band structure and bandgap values, effective mass of electrons and holes, work function and conduction and valence band edge potentials of BSe and M_2_CO_2_ (M = Ti, Zr, Hf) monolayers are in agreement with previously available data. Binding energies, interlayer distance and *Ab initio* molecular dynamic simulations (AIMD) calculations show that BSe–M_2_CO_2_ (M = Ti, Zr, Hf) vdW heterostructures are stable with specific stacking and demonstrate that these heterostructures might be synthesized in the laboratory. The electronic band structure shows that all the studied vdW heterostructures have indirect bandgap nature – with the CBM and VBM at the *Γ*–*K* and *Γ*-point of BZ for BSe–Ti_2_CO_2_, respectively; while for BSe–Zr_2_CO_2_ and BSe–Hf_2_CO_2_ vdW heterostructures the CBM and VBM lie at the *K*-point and *Γ*-point of BZ, respectively. Type-II band alignment in BSe–M_2_CO_2_ (M = Ti, Zr, Hf) vdW heterostructures prevent the recombination of electron–hole pairs, and hence are crucial for light harvesting and detection. Absorption spectra are investigated to understand the optical behavior of BSe–M_2_CO_2_ (M = Ti, Zr, Hf) vdW heterostructures, where the lowest energy transitions are dominated by excitons. Furthermore, BSe–M_2_CO_2_ (M = Ti, Zr, Hf) vdW heterostructures are found to be potential photocatalysts for water splitting at pH = 0, and exhibit enhanced optical properties in the visible light zones.

## Abstract 2

Using density functional theory calculations, we have investigated the electronic band structure, optical and photocatalytic response of BSe, M_2_CO_2_ (M = Ti, Zr, Hf) monolayers and their corresponding BSe–M_2_CO_2_ (M = Ti, Zr, Hf) van der Waals heterostructures.

## Graphical abstract

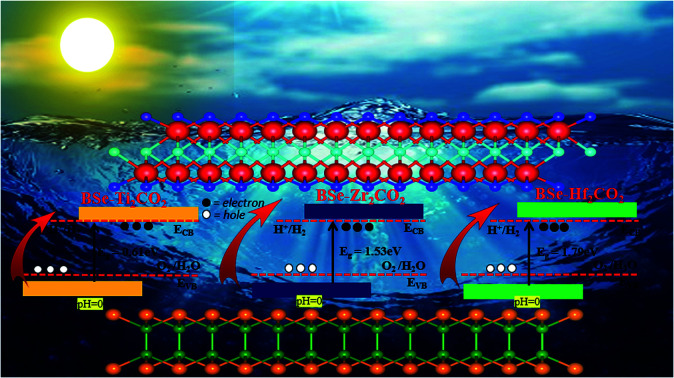

## 1. Introduction

After the successful synthesis of graphene,^1–4^ great attention has been paid to other 2D materials, such as hexagonal boron nitrides (h-BN),^5^ blue and black phosphorene,^6^ transition metal dichalcogenides (TMDCs),^7^ silicene,^8^ germanene,^9^ MXenes,^10^ and Janus transition metal dichalcogenides (JTMDCs).^11^ Among these materials, MXenes (M_*n*+1_X_*n*_), synthesized by eliminating the A-layer from their bulk counterpart the MAX phase (M_*n*+1_AX_*n*_, M refers to early transition metals, “A” represents the group of sp elements, “X” represents C or N atoms, and *n* is 1, 2, 3), has received wide research attention^12^ due to a wide range of applications in Li-ion batteries,^13^ catalysis,^14^ electrochemical capacitors^15^ and also in fuel cells.^16^ The M–X bond in the MAX crystals is stronger than the M–A bond, making it possible to etch “A” atoms between the M_*n*+1_X_*n*_ layer.^17^ All the MXenes are metals, while appropriate surface termination (M_*n*+1_X_*n*_T_*x*_, T_*x*_ denotes surface terminations, *i.e.* O, F, OH) makes them semiconductors.^18^

Tuning the properties of 2D materials has led to a new field that assembles 2D materials (isolated) into hybrid heterostructures in a precisely controlled sequence of layer by layer stacking, called vdW heterostructures.^19^ It provides a versatile platform for exploring new phenomena and designing novel nanoelectronic devices.^20,21^ To date, a great deal of vdW heterostructures have been studied theoretically^22–27^ and perceived experimentally.^28–31^ These vdW heterostructures are also utilized to create electronic and optoelectronic devices with novel physical properties and applications.^32–37^

MXenes-based vdW heterostructures, such as MXenes–MXenes,^38^ MXene and nitrogen-doped graphene,^39^ MXenes–TMDCs,^40^ MXene–blue phosphorene,^41^ MXenes and B-doped graphene,^42^ have already been fabricated and investigated in detail. BSe, another 2D material, has been proposed and predicted to be thermally stable with indirect bandgap nature.^43,44^

Motivated by the fascinating optoelectronic and photocatalytic performance of MXenes with other monolayers in the form of vdW heterostructures, we have fabricated BSe–M_2_CO_2_ (M = Ti, Zr, Hf) vdW heterostructures. Indeed small lattice mismatch and the same hexagonal symmetry of the BSe and M_2_CO_2_ (M = Ti, Zr, Hf) monolayer allow the creation of BSe–M_2_CO_2_ (M = Ti, Zr, Hf) vdW heterostructures. It is also surprising that there is no previous work on the BSe–M_2_CO_2_ (M = Ti, Zr, Hf) vdW heterostructures. We have investigated the structural and electronic properties, band alignments, average and planar electrostatic potentials, Bader charge analysis, optical and photocatalytic response of BSe, M_2_CO_2_ (M = Ti, Zr and Hf) monolayers and their vdW heterostructure. Our results show that BSe–M_2_CO_2_ (M = Ti, Zr) vdW heterostructures are a promising novel material for visible light photocatalysis, electronic and optoelectronic devices.

## 2. Computational details

We used DFT^45^ with empirical dispersion correction of Grimme^46^ and Perdew–Burke–Ernzerhof (PBE)^47^ functional in Vienna *ab initio* simulation package (VASP).^48,49^ In the first Brillouin zone, a *Γ*-point centered 6 × 6 × 1 Monkhorst–Pack *k*-point grid and 500 eV cutoff energy were used. A vacuum layer thickness of 25 Å is established to avoid the interaction of the adjacent layers of atoms. The geometric relaxations are carried out until we achieve the convergence criterion of 10^−4^ eV Å^−1^ (10^−5^ eV) for forces (energy). Commonly, the PBE functional underestimates the band gap values of semiconductors, therefore, we have also performed a computationally expensive HSE06 (Heyd–Scuseria–Ernzerhof)^50^ functional for the precise calculation of the electronic structure and band gap values.

*Ab initio* molecular dynamic simulations (AIMD)^51^ are used to investigate the thermal stabilities of BSe–M_2_CO_2_ (M = Ti, Zr) vdW heterostructures. AIMD simulations are performed through the Nose thermostat algorithm at a temperature of 300 K for a total of 6 ps with a time interval of 1 fs.

Furthermore, we have solved the Bethe–Salpeter equation (BSE) in GW calculations using the Quantum-Espresso program package,^52^ to explore the optical spectra estimated by the imaginary part of the dielectric function (*ε*_2_(*ω*)) of the BSe–M_2_CO_2_ (M = Ti, Zr, Hf) vdW heterostructures.^53–55^

## 3. Results and discussion

**Fig. 1:**
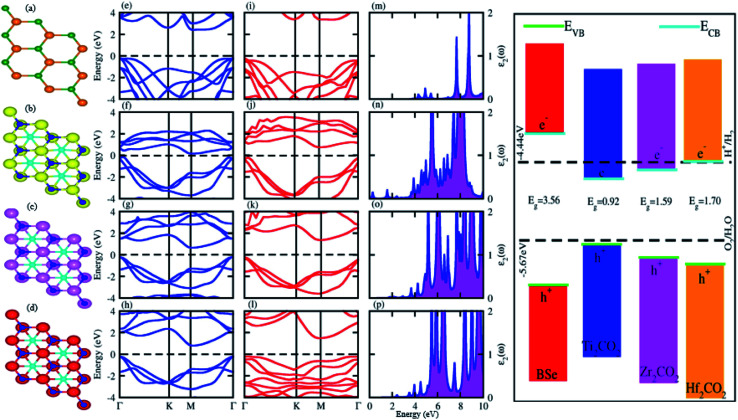
Geometrical structure (top view), electronic band structure (PBE(blue), HSE06(red)), and imaginary part of dielectric function (*ε*_2_(*ω*)), of BSe ((a), (e), (i) and (m)), Ti_2_CO_2_ ((b), (f), (j) and (n)), Zr_2_CO_2_ ((c), (g), (k) and (o)), and Hf_2_CO_2_ ((d), (h), (l) and (p)), and their photocatalytic response.

Optimized lattice constant, bond length, bandgap values, effective mass of electrons and holes, work function and conduction and valence band edge potentials (*E*_CB_ and *E*_VB_) of BSe and M_2_CO_2_ (M = Ti, Zr, Hf) monolayers in Table 1, are in agreement with ref. 56–58. Optimized geometry (top view) and electronic band structure (using PBE and HSE06 functional) are presented in Fig. 1, and show that both BSe and M_2_CO_2_ (M = Ti, Zr, Hf) monolayers are indirect bandgap semiconductors with CBM(VBM) at the M(*Γ*)-point of BZ. The calculated effective mass for both holes and electrons in Table 1, show that BSe and Hf_2_CO_2_ monolayers would have high carrier mobility.^59^ Difference in the work functions in Table 1, show that in the case of the interface of these materials, electrons will spontaneously flow from M_2_CO_2_ to the BSe monolayer, which is further explained in detail later in the vdW heterostructure of BSe and M_2_CO_2_ (M = Ti, Zr, Hf) monolayers.^60^ Furthermore, the imaginary part of the dielectric function in Fig. 1, shows that the first excitonic peak at 3.851 for BSe, 0.286 for Ti_2_CO_2_, 1.79 for Zr_2_CO_2_, and 2.416 eV for the Hf_2_CO_2_ monolayer, lies in the visible range of the spectrum, consistent with ref. 61–63. In the case of the photocatalytic response at pH = 0, BSe and Hf_2_CO_2_ cross both the conduction and valence band edge potentials, while Ti_2_CO_2_ and Zr_2_CO_2_ cross the valence band edge potential only and fail to cross the conduction band edge, in agreement with ref. 56, 59 and 64, hence showing the potential of these systems in electronic, optoelectronic and photocatalytic applications. The above discussed consistencies for BSe and M_2_CO_2_ (M = Ti, Zr, Hf) monolayers, show the authenticity of the present approach for the calculation of BSe–M_2_CO_2_ (M = Ti, Zr, Hf) vdW heterostructures.

**Table 1:** Lattice constant (*a* in Å), bond length (B–Se, M–O and M–C in Å), band gap (*E*_g_ in eV), effective mass (
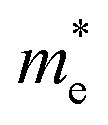
 and 
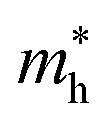
), work function (*ϕ* in eV) and conduction and valence band edge potentials (*E*_CB_ and *E*_VB_ in eV) for BSe monolayer and M_2_CO_2_ (M = Ti, Zr, Hf) MXenes

Monolayers	BSe	Ti_2_CO_2_	Zr_2_CO_2_	Hf_2_CO_2_
*a*	3.26	3.01	3.31	3.27
B–Se	2.10	—	—	—
M–O	—	1.970	2.119	2.091
M–C	—	2.210	2.359	2.332
*E*_g-PBE_	2.635	0.300	0.865	0.99
*E*_g-HSE06_	3.56	0.920	1.590	1.70
d1ra07569a-t3	0.42	0.87	0.69	0.61
d1ra07569a-t3	0.93	1.32	1.05	1.27
*Φ*	3.953	5.536	4.835	4.450
*E*_CB_	−1.255	0.354	0.069	−0.005
*E*_VB_	2.304	1.248	1.659	1.695

**Fig. 2:**
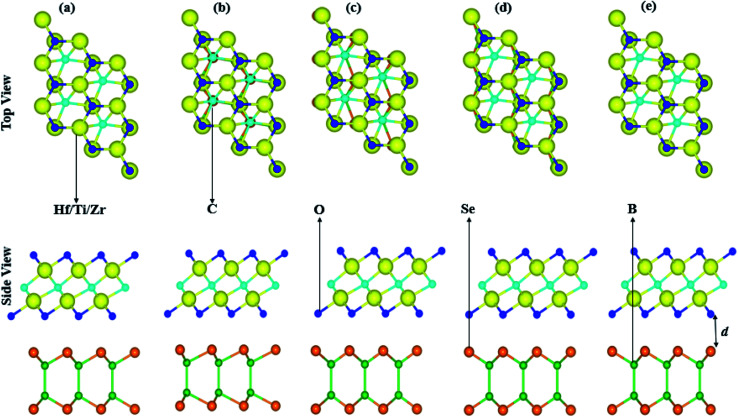
Possible stacking configurations of the BSe–M_2_CO_2_ (M = Ti, Zr, Hf) van der Waal heterostructures.

Lattice mismatch of BSe, with Ti_2_CO_2_ of 4.9%, with Zr_2_CO_2_ of 1.2% and with Hf_2_CO_2_ of 0.03%, are experimentally achievable^65^ and the same hexagonal symmetry realizes the fabrication of BSe–M_2_CO_2_ (M = Ti, Zr, Hf) vdW heterostructures. The electronic band structure is very sensitive to layer stacking,^66^ therefore we have chosen five possible stacking configurations of BSe–M_2_CO_2_ (M = Ti, Zr, Hf) vdW heterostructures, see Fig. 2. In stacking (a) the M(O) atom of M_2_CO_2_ is placed on top of the Se(B) atom of the BSe monolayer; in stacking (b) the M(C) atom of M_2_CO_2_ is placed on top of the B(Se) atom of the BSe monolayer; in stacking (c) the O(C) atom of M_2_CO_2_ is placed on top of the Se(B) atom of the BSe monolayer; in stacking (d) the O(M) atom of M_2_CO_2_ is placed on top of the Se(B) atom of the BSe monolayer; and in stacking (e) the O(M) atom of M_2_CO_2_ is placed on top of the (B) atom of the BSe monolayer, while the C is on a hexagonal site.

Binding energy; *E*_b_ = *E*_BSe–M_2_CO_2__ − *E*_M_2_CO_2__ − *E*_BSe_, where *E*_BSe–M_2_CO_2__ is the total energy of the BSe–M_2_CO_2_ (M = Ti, Zr, Hf) vdW heterostructure, *E*_M_2_CO_2__ is the total energy of the isolated M_2_CO_2_ (M = Ti, Zr, Hf) MXene, and *E*_BSe_ is the total energy of the isolated BSe monolayer along with interlayer distance of the stacking as presented in Table 2. Smaller interlayer distance and binding energies represent the most stable stacking configuration, therefore, stacking (a) of the BSe–M_2_CO_2_ (M = Ti, Zr, Hf) vdW heterostructures is the most stable configuration. Obviously, negative binding energies show that the formation of all heterostructures are exothermic, see Table 2. These values are in the range of binding energies for other vdW heterostructures,^67,68^ hence suggest the possible experimental fabrication of BSe–M_2_CO_2_ vdW heterostructures. The calculated interlayer distance (see Table 2) also confirms weak vdW interactions in the stacked layers of these heterostructures. Optimized lattice constants of the most stable stacking configurations are presented in Table 3.

**Table 2:** Binding energies (*E*_b_ in eV) and inter layer distance (*d* in Å) of the BSe–M_2_CO_2_ (M = Ti, Zr, Hf) vdW heterostructures in different stacking configurations

Stacking	BSe–Ti_2_CO_2_	BSe–Zr_2_CO_2_	BSe–Hf_2_CO_2_
*E*_b_ (a)	−0.429	−0.395	−0.297
*d*	3.33	3.32	3.33
*E*_b_ (b)	−0.326	−0.316	−0.268
*d*	3.42	3.41	3.39
*E*_b_ (c)	−0.331	−0.337	−0.284
*d*	3.39	3.38	3.35
*E*_b_ (d)	−0.409	−0.305	−0.277
*d*	3.37	3.41	3.39
*E*_b_ (e)	−0.398	−0.327	−0.281
*d*	3.46	3.39	3.36

**Table 3:** Lattice constant (in Å), bandgap values (*E*_g_ in eV), effective mass (
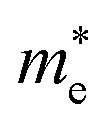
 and 
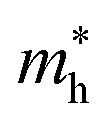
), work function (*ϕ* in eV), potential difference (Δ*V*) conduction and valence band edges (*E*_VB_ and *E*_CB_ in eV) of BSe–M_2_CO_2_ (M = Ti, Zr, Hf) vdW heterostructures

Heterostructure	BSe–Ti_2_CO_2_	BSe–Zr_2_CO_2_	BSe–Hf_2_CO_2_
*a*	3.15	3.29	3.27
*E*_g-PBE_	0.107	0.837	0.970
*E*_g-HSE06_	0.61	1.536	1.79
Δ*V*	4.280	2.300	2.050
*ϕ*	6.537	5.764	5.808
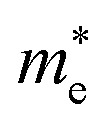	0.39	0.73	0.51
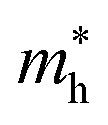	0.76	1.08	0.97
*E*_VB_	0.477	−0.0046	−0.0617
*E*_CB_	1.0876	1.575	1.657

**Fig. 3:**
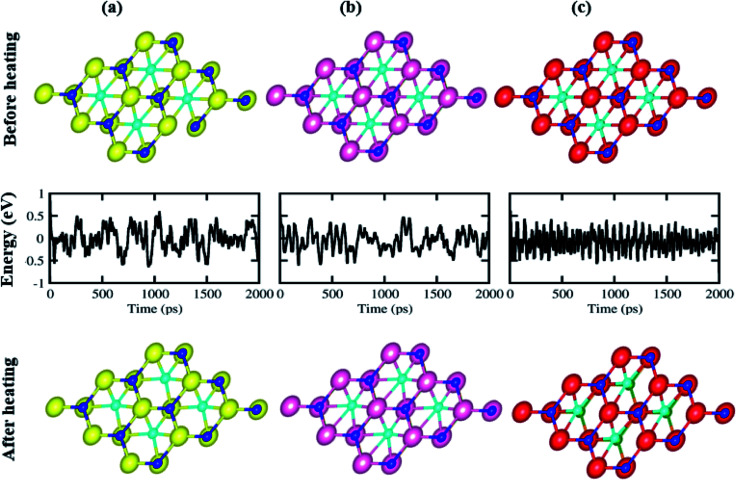
Geometrical structure before heating (first row), with fluctuating energy (second row) and after heating (third row) of: (a) BSe–Ti_2_CO_2_, (b) BSe–Zr_2_CO_2_, and (c) BSe–Hf_2_CO_2_ vdW heterostructures using AIMD simulation.

To further verify the thermal stability of the stacking of (a) BSe–M_2_CO_2_ (M = Ti, Zr, Hf) vdW heterostructures, we have used the AIMD simulation. We have chosen a 3 × 3 supercell with top view, see Fig. 3. It is clear from the figure that after heating for 5 ps at 1 fs time steps at 300 K, the BSe–M_2_CO_2_ (M = Ti, Zr, Hf) vdW heterostructures show no broken bonds (remain stable), while the free energy oscillates slightly (see Fig. 3, middle row), which confirms the thermal stability of these systems at 300 K. Therefore, the stacking of the (a) BSe–M_2_CO_2_ (M = Ti, Zr, Hf) vdW heterostructures is the most stable structure configuration and will be further examined in detail.

**Fig. 4:**
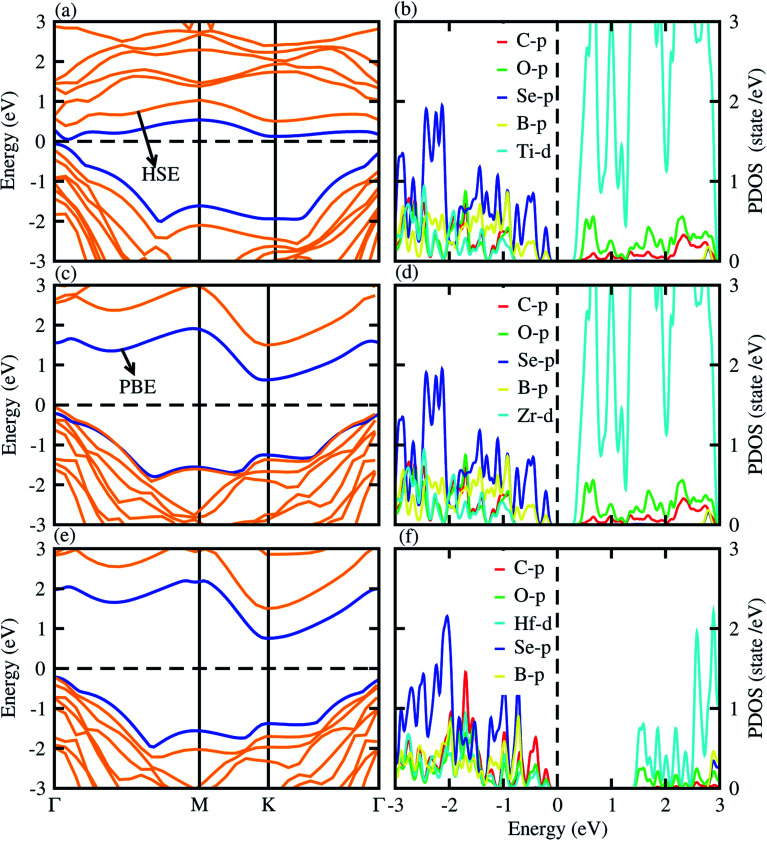
PBE (blue) and HSE06 (yellow) band structures (left column) and partial density of states (right column) of the BSe–Ti_2_CO_2_ ((a) and (b)), BSe–Zr_2_CO_2_ ((c) and (d)), BSe–Hf_2_CO_2_ ((e) and (f)) vdW heterostructures.

Using both PBE and HSE06 functionals, we have calculated the electronic band structures of BSe–M_2_CO_2_ (M = Ti, Zr, Hf) vdW heterostructures, see Fig. 4, while the calculated bandgap values are presented in Table 3. The electronic band structure shows that all the studied vdW heterostructures have an indirect band nature with the CBM and VBM at the *Γ*–*K* and *Γ*-point of BZ for BSe–Ti_2_CO_2_, (see Fig. 4(a)), while both BSe–Zr_2_CO_2_ and BSe–Hf_2_CO_2_ vdW heterostructures are indirect bandgap semiconductors with CBM at the *K*-point and VBM at the *Γ*-point of the first BZ (see Fig. 4(b) and (c)). In the case of the BSe–Ti_2_CO_2_ vdW heterostructure direct recombination of photogenerated electrons and holes hence play a crucial rule in optoelectronic devices.^69^ In the case of the BSe–Zr_2_CO_2_ and BSe–Hf_2_CO_2_ vdW heterostructures, the recombination of photogenerated electrons and holes is slow because firstly the CBM and VBM momenta align themselves and then recombination occurs, which is useful for laser applications.^70–72^ The variation in bandgap values (given in Table 3) and the band structures of BSe–M_2_CO_2_ (M = Ti, Zr, Hf) vdW heterostructures from their parent monolayers, reveals the bandgap engineering making the vdW heterostructures.^73^ The contribution of the different atomic states to the Fermi level is further explored by investigating the partial density of states (PDOS), see Fig. 4 (b), (d) and (f). One can see that the CBM is mainly due to the d state of Ti/Zr/Hf atoms of the M_2_CO_2_ layer, while the VBM is due to the p state of the Se atom of BSe layer.

**Fig. 5:**
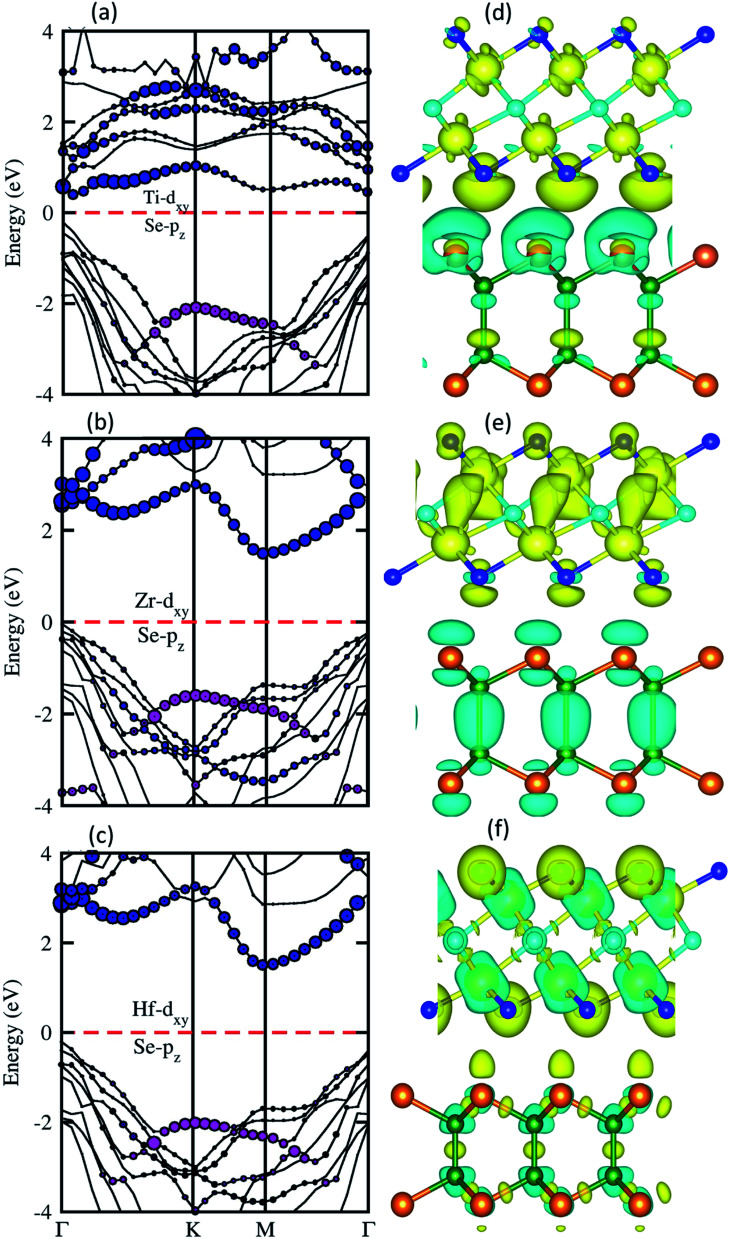
Weighted band structure (left column) and 3D isosurface 0.001 eV Å ^−3^ of the charge density difference (right column) of BSe–Ti_2_CO_2_ ((a) and (d)), BSe–Zr_2_CO_2_ ((b) and (e)) and BSe–Hf_2_CO_2_ ((c) and (f)) vdW heterostructures. The cyan(yellow) color shows the charge electrons depletion(accumulation).

To verify the contribution of different atomic states in the VBM and CBM, and nature of the band structure for type-I and type-II, we have calculated the weighted band structure of BSe–M_2_CO_2_ (M = Ti, Zr, Hf) vdW heterostructures, plotted in Fig. 5. One can clearly see that in the case of BSe–Ti_2_CO_2_ vdW heterostructures (Fig. 5(a)) at the *Γ*-point of BZ, the main contribution in the CBM is due to the Ti-d_*xy*_ atom of Ti_2_CO_2_ monolayers while the VBM is due to the Se-p_*xy*_ state of BSe monolayers, hence confirming type-II band alignment.^74,75^ In the case of the BSe–Zr_2_CO_2_ and BSe–Hf_2_CO_2_ vdW heterostructures (see Fig. 5(b) and (c), respectively) the main contribution in the CBM(VBM) is due to the Zr/Hf-d_*xy*_ (Se-p_*xy*_) states of the Zr_2_CO_2_, Hf_2_CO_2_ (BSe) monolayers at the K(*Γ*)-point of BZ, which also shows type-II band alignment. The localization of the VBM and CBM from different layers are obtained without any external electric field, as the intrinsic electric field induces bond bending in making the vdW heterostructures.^76,77^ This induced field drive photogenerated electrons and holes in different directions. Type-II band alignment is an effective tool to enhance electron–holes pairs which reduce the recombination time, applicable for light harvesting and detection.^74,75^ The spontaneous apprehension about the charge transfer is obtained from the deportation charge density (DCD) isosurface, presented in Fig. 5(d–f) for BSe–M_2_CO_2_ (M = Ti, Zr, Hf) vdW heterostructures. In Fig. 5(d–f) the cyan(yellow) color shows the charge electrons depletion(accumulation), hence confirming that charge is transferred from M_2_CO_2_ (M = Ti, Zr, Hf) to BSe monolayers at the interface of the BSe–M_2_CO_2_ vdW heterostructures, which leads to p-doping in Ti_2_CO_2_, Zr_2_CO_2_ and Hf_2_CO_2,_ and n-doping in the BSe monolayer. For further verification and quantification of charge transfer we have investigated the Bader charge analysis, which shows that the charge of about 0.17, 0.09 and 0.11 e/unitcell is transferred from the Ti_2_CO_2_, Zr_2_CO_2_ and Hf_2_CO_2_ to the BSe monolayer, respectively.^78^ This transfer of charge confirms that due to long range vdW forces, the interlayer bonding of Ti_2_CO_2_, Zr_2_CO_2_, Hf_2_CO_2_ and BSe monolayers can be weak and diminishes with increasing bond length.

**Fig. 6:**
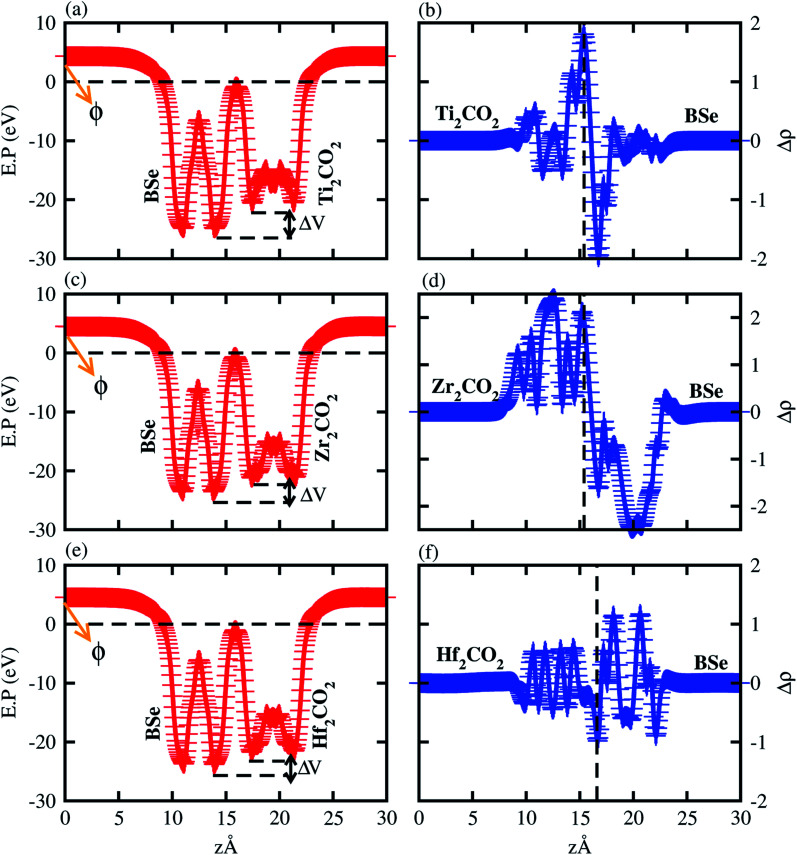
Average and planar electrostatic potential of (a and b) BAse–Ti_2_CO_2_, (c and d) BSe–Zr_2_CO_2_ and (e and f) BSe–Hf_2_CO_2_. The work function (*ϕ*) and potential drop (Δ*V*) are highlighted.

Furthermore, we have verified the transfer of charge and potential difference by calculating the average and planar electrostatic potential difference along the *z*-axis, see Fig. 6. One can easily see that the BSe monolayer has a deeper potential then Ti_2_CO_2_, Zr_2_CO_2_ and Hf_2_CO_2_ monolayers in BSe–M_2_CO_2_ vdW heterostructures (see Fig. 6), confirming the transfer of charge from Ti_2_CO_2_, Zr_2_CO_2_ and Hf_2_CO_2_ to the BSe layer. Also, the potential drop (DV) across the vdW heterostructures, given in Table 3, facilitates the separation of electrons and holes at the interface. Making vdW heterostructures may effect the work function, which leads to enhanced electronic properties of the vdW heterostructures. Therefore, we have calculated the work function of monolayers and their vdW heterostructures, as presented in Table 1 and 3. One can easily see that the work function of vdW heterostructures is almost the average of the corresponding monolayers, efficient for charge transfer.

Furthermore, we have calculated the effective mass of electrons and holes in the BSe–M_2_CO_2_ vdW heterostructures. Smaller effective mass leads to higher carrier mobility which is useful for high performance nanoelectronic devices.^79^ We used parabolic fitting for the VBM and CBM and investigated the effective mass of electrons and holes of the BSe–M_2_CO_2_ vdW heterostructures. The value for effective mass of the holes and electrons are given in Table 3. One can see that the effective mass of vdW heterostructures (for holes and electrons) is smaller than that of the corresponding monolayers in Table 1, hence are suitable for application in high-performance nanoelectronic devices.

**Fig. 7:**
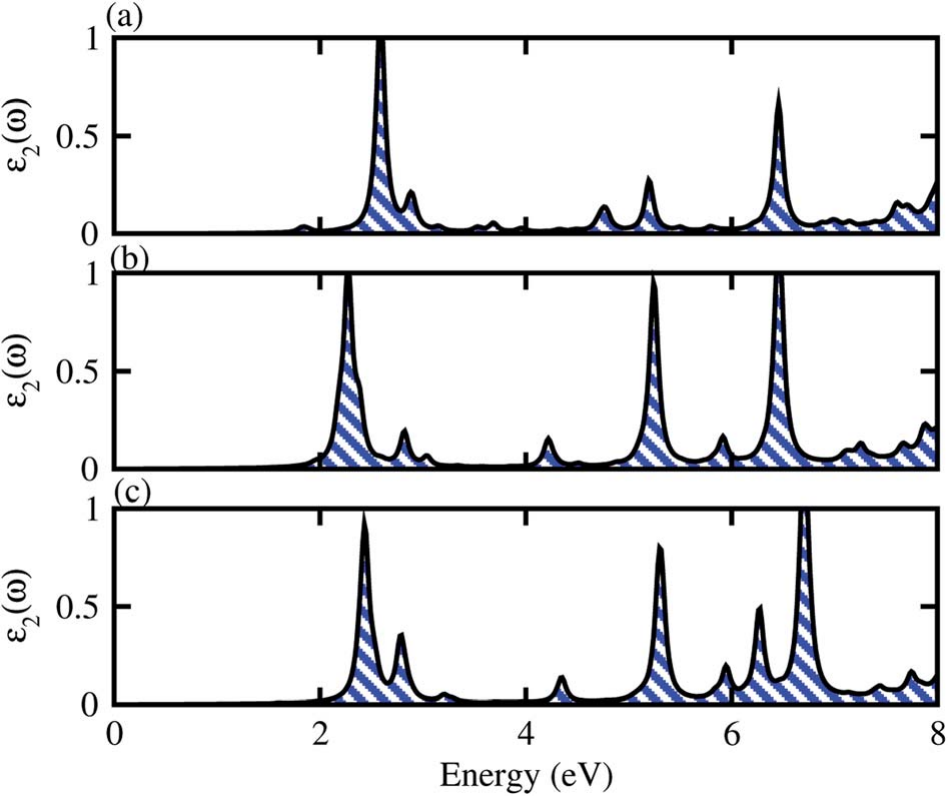
Optical absorption of (a) BSe–Ti_2_CO_2_, (b) BSe–Zr_2_CO_2_ and (c) BSe–Hf_2_CO_2_.

We have also calculated the optical performance in terms of imaginary parts of the dielectric function (*ε*_2_(*ω*)) of BSe–M_2_CO_2_ (M = Ti, Zr, Hf) vdW heterostructures as a function of photon energy, given in Fig. 7. One can see that optical transitions are dominated by excitons at 2.59 eV for Ti_2_CO_2_, at 2.27 eV for Zr_2_CO_2_ and at 2.43 eV for Hf_2_CO_2_. The calculated exciton binding energies are 0.77, 0.048 and 0.143, respectively (see Fig. 7). All these BSe–M_2_CO_2_ (M = Ti, Zr, Hf) vdW heterostructures show substantial absorption in visible and UV regions of the spectrum. This can be attributed to the fact that the charge transfer and interlayer coupling, which can result in the overlap of electronic states in the valence bands of the heterostructure, and which enhances the optical absorption (see Fig. 1 and 7).^80–82^

**Fig. 8:**
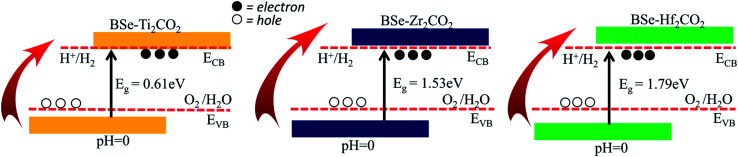
Band alignment for the valence band (VB) and conduction band (CB) edge of BSe–Ti_2_CO_2_, BSe–Zr_2_CO_2_ and BSe–Hf_2_CO_2_, at pH = 0. The standard oxidation (−5.67 eV, O_2_/H_2_O) and reduction (−4.44 eV, H^+^/H^2^) potentials are also labeled.

We have also investigated the photocatalytic^83–86^ properties of BSe–M_2_CO_2_ (M = Ti, Zr, Hf) vdW heterostructures using the Mulliken electronegativity.^87,88^ Appropriate bandgap size, valence and conduction band edges must straddle the redox potentials of water, as reported in our previous work^89^ for use in the water splitting reaction. The standard water redox potentials are −4.50 eV for the reduction (H^+^/H_2_) and −5.73 eV for the oxidation (H_2_O/O_2_).^90^ The calculated band edge potentials *E*_VBM_ and *E*_CBM_ of the heterostructures by the HSE06 functional are summarized in Table 3 and presented in Fig. 8. Valence band edge potential and conduction band edge potential, (*E*_VBM_ and *E*_CBM_) for BSe–Hf_2_CO_2_ and BSe–Zr_2_CO_2_ vdW heterostructures are higher than that of H^+^/H_2_ and H_2_O/O_2_. These results signify that, BSe–Hf_2_CO_2_ and BSe–Zr_2_CO_2_ vdW heterostructures can oxidize H_2_O/O_2_ and reduce H^+^/H_2_,^90^ which is suitable for the production of clean and renewable energy equipment applications.^91^ Although, the Zr_2_CO_2_ monolayer fails to oxidize water (see Fig. 1 and Table 1), the BSe–Zr_2_CO_2_ vdW heterostructure shows a good response to water redox potential, hence making the vdW heterostructure suitable for the production of clean and renewable energy device applications.^91^ Similar to the corresponding monolayer, in the case of BSe–Ti_2_CO_2_, the *E*_VB_(*E*_CB_) cross(fail to cross) the reduction level. All these findings demonstrate that the BSe–M_2_CO_2_ heterostructures can be considered as potential photocatalysts for water splitting and provide theoretical guidance for designing high-performance nano-electronic and optoelectronic devices based on the BSe–M_2_CO_2_ heterostructures.^92–94^

## 4. Conclusion

In summery, using first principles DFT calculations, we have investigated the electronic band structure, optical and photocatalytic response of BSe, M_2_CO_2_ (M = Ti, Zr, Hf) monolayers and their corresponding BSe–M_2_CO_2_ (M = Ti, Zr, Hf) vdW heterostructures. The calculated lattice parameters, electronic band structure, bandgap values and valence and conduction band edge potentials of BSe and M_2_CO_2_ (M = Ti, Zr, Hf) monolayers are in good agreement with previous available data, showing the authenticity of the present approach for the calculations of BSe–M_2_CO_2_ (M = Ti, Zr, Hf) vdW heterostructures. Based on the binding energy and interlayer distance calculations, stacking (a) of the five different stacking of BSe–M_2_CO_2_ (M = Ti, Zr, Hf) vdW heterostructures is the most stable stacking configuration. Furthermore, AIMD simulations also show that stacking (a) for all studied systems, is thermally stable at 300 K. Surprisingly, in contrast to the parent monolayers, BSe–Ti_2_CO_2_ (BSe–Zr_2_CO_2_ and BSe–Hf_2_CO_2_) vdW heterostructures are direct (indirect) band gap semiconductor(s). All studied vdW heterostructures have type-II band alignment, hence play a major role in light harvesting and detection. Bader charge analysis shows transfer of charge from M_2_CO_2_ (M = Ti, Zr, Hf) to the BSe layer, hence N(P)-type doping is achieved in the M_2_CO_2_(BSe) monolayer at the interface of BSe–M_2_CO_2_ vdW heterostructures. The imaginary part of the dielectric function is also investigated to understand the optical absorption of BSe–M_2_CO_2_ (M = Ti, Zr, Hf) vdW heterostructures, where the lowest energy transitions are dominated by excitons. The calculated photocatalytic response signifying that BSe–Zr_2_CO_2_ and BSe–Hf_2_CO_2_ vdW heterostructures can oxidized H_2_O/O_2_ and reduce H^+^/H_2_, while the Zr_2_CO_2_ monolayer fails to oxidize water, hence making BSe–M_2_CO_2_ vdW heterostructures viable for the production of clean and renewable energy device applications. Similar to the corresponding monolayer, in the case of BSe–Ti_2_CO_2_, the *E*_VBM_(*E*_CBM_) cross(fail to cross) the reduction level.

## Conflicts of interest

There are no conflicts to declare.

## Supplementary Material
